# Random epigenetic modulation of CHO cells by repeated knockdown of DNA methyltransferases increases population diversity and enables sorting of cells with higher production capacities

**DOI:** 10.1002/bit.27493

**Published:** 2020-07-24

**Authors:** Marcus Weinguny, Peter Eisenhut, Gerald Klanert, Nikolaus Virgolini, Nicolas Marx, Andreas Jonsson, Daniel Ivansson, Ann Lövgren, Nicole Borth

**Affiliations:** ^1^ ACIB—Austrian Centre of Industrial Biotechnology Graz Austria; ^2^ Department of Biotechnology University of Natural Resources and Life Sciences Vienna Austria; ^3^ Cytiva Uppsala Sweden

**Keywords:** cell line development, CHO cells, DNA methylation, epigenetic modulation, productivity improvement

## Abstract

Chinese hamster ovary (CHO) cells produce a large share of today's biopharmaceuticals. Still, the generation of satisfactory producer cell lines is a tedious undertaking. Recently, it was found that CHO cells, when exposed to new environmental conditions, modify their epigenome, suggesting that cells adapt their gene expression pattern to handle new challenges. The major aim of the present study was to employ artificially induced, random changes in the DNA‐methylation pattern of CHO cells to diversify cell populations and consequently increase the finding of cell lines with improved cellular characteristics. To achieve this, DNA methyltransferases and/or the ten‐eleven translocation enzymes were downregulated by RNA interference over a time span of ∼16 days. Methylation analysis of the resulting cell pools revealed that the knockdown of DNA methyltransferases was highly effective in randomly demethylating the genome. The same approach, when applied to stable CHO producer cells resulted in (a) an increased productivity diversity in the cell population, and (b) a higher number of outliers within the population, which resulted in higher specific productivity and titer in the sorted cells. These findings suggest that epigenetics play a previously underestimated, but actually important role in defining the overall cellular behavior of production clones.

## Graphical abstract

Weinguny and coworkers demonstrate that overall DNA methylation patterns in Chinese hamster ovary (CHO) cells can effectively be randomized by a knock‐down of DNA methyltransferases (DNMT) using small interfering (si)RNAs. These DNA methylation changes increase the phenotypic diversity in a CHO cell population producing a recombinant protein. This newly generated diversity eventually facilitated the isolation of cells with increased production capacities and highlights the importance of epigenetic regulation and its impact on the phenotype of mammalian cell factories.

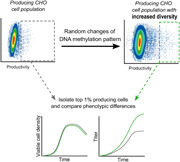

**Abbreviations**
*µ*: growth rate
CHO: Chinese hamster ovary (cells)
CLD: cell line development
CV, coefficient of variation; DMR: differently methylated regions
DNMT: DNA methyltransferases
EPO‐FC: erythropoietin–fragment crystallizable fusion protein
GOI: gene of interest
mock: H_2_0 control
qP: specific productivity
Res: resveratrol
RNAi: RNA interference
TET: ten‐eleven translocation enzyme
scram: scramble siRNA
siRNA: small interfering RNA
VCD: viable cell density; WGBS, whole genome bisulfite sequencing

## 1 INTRODUCTION

Chinese hamster ovary (CHO) cells are one of the most widely used expression systems for recombinant production of therapeutic proteins (Walsh, 2018). Although these cells are equipped with the necessary factors and machinery to produce the desired product for safe use in humans, there is high diversity in phenotypes between cell lines and subclones, so that extensive screening of many thousand clones is often required for the generation of a good and stable production cell line (Pilbrough, Munro, & Gray, 2009). Cell line development (CLD) processes are often labor‐intensive and/or need to be supported by expensive automation or robotics (Mocciaro et al., 2018). The precise setting of different parameters in the CLD platform, such as the medium used during subcloning, the culture conditions (e.g., static microtiter plates vs. shaken deep well plates), the selective agent or other selection tools used (e.g., cell sorting) have an impact on the clones that are generated. These factors may contribute to the high diversity in clone behavior that is observed even between the clones from the same pipeline or between subclones derived from an already subcloned cell line. Many studies that investigated transcriptomic, proteomic, or other phenotypic traits of high‐ and low‐producing CHO cell lines identified large numbers of differentially regulated genes, so‐called “engineering targets” (Nissom et al., 2006; Orellana et al., 2015). Intriguingly, rationally targeting, for example, by transgenic overexpression of these genes, often does not impact productivity as predicted, or enhances it by different routes (Orellana, Marcellin, Gray, & Nielsen, 2017). Another screening of transcriptomic differences between high‐ and low‐producing CHO cell lines revealed no impact of the gene copy number but over 600 differently expressed genes involved, among others, in crucial cellular processes such as transcription and protein transport were identified (Chen et al., 2019). However, there was little overlap in the specific differentially expressed genes, although in many cases similar pathways were shown to be differentially expressed. In addition, Vishwanathan et al. (2014) showed that it is not only the increasing copy number of the gene of interest (GOI) during gene amplification that is responsible for higher productivities but also a stepwise change and adaptation of the cell's transcriptome pattern that occurs during the exposure to the selection agents and allows cells to handle higher production challenges. These studies and many others indicate that whether a cell is a high performer or not depends to a large degree on the precise regulation of many genes that contribute to productivity and the fine‐tuned combination of their expression levels (Harreither et al., 2015; Tamošaitis & Smales, 2018).

The gene expression pattern in mammalian cells is controlled on many different layers, for example, by direct control of transcription rate via transcription factors (Amini et al., 2019; Gutiérrez‐González et al., 2019), by epigenetic marks around coding regions (Gibney & Nolan, 2010; Wippermann, Rupp, Brinkrolf, Hoffrogge, & Noll, 2015) or by microRNA control of messenger RNA (mRNA) levels (Carthew & Sontheimer, 2009; Wahid, Shehzad, Khan, & Kim, 2010). Epigenetics describes mechanisms in a cell by which the expression of genes is controlled by covalent modifications of DNA, RNA, or histones or by small and long noncoding RNAs that, importantly, leave the genetic code itself unaltered (Gibney & Nolan, 2010). Such covalent modifications comprise for example, methylation of cytosine in the DNA (the so‐called methylome) or the methylation or acetylation of lysines in the N‐terminal tails of histones. Where and/or in which combination these modifications are present in different genomic regions, for example, around transcription start sites, in promoter or enhancer regions, determines whether a gene is actively expressed or silenced (Attwood, Yung, & Richardson, 2002; Hu et al., 2016; Razin & Cedar, 1991). The key players that control and interpret these marks are the so‐called epigenetic readers, writers, and erasers. Each group comprises a set of many different enzymes such as the DNA methyltransferases (DNMTs), ten‐eleven translocation (TET) enzymes, histone acetylase transferases, histone deacetylases (HDACs), and many more (Biswas & Rao, 2018). Conceivably, these enzymes represent interesting targets to induce changes in a cell's gene expression pattern.

Changing epigenetic marks can be accomplished in two ways, either randomly or by a targeted approach. Targeted epigenetic engineering has recently been developed and usually links an epigenetic modifier domain (e.g., DNMT3a) to a guiding factor, in most cases today a catalytically inactive Cas9 enzyme that directs the complex to the correct genomic locus. Thus one can rationally and reversibly switch on or off the expression of endogenous genes solely by changing the promoter DNA methylation of the target region (Marx et al., 2018; Morita et al., 2016; O'Geen et al., 2017). While many other approaches, such as the targeting of transcription factors (Agne et al., 2014; Karottki et al., 2020) typically are transient, changes in DNA methylation are maintained and inherited by progeny cells over many generations (Marx et al., 2018). The main limitation of this approach is that only genes with an already known impact on productivity or phenotype and only a limited number of genes can be targeted and the effect of targeting promoter regions is predominantly on/off, but does not achieve fine tuning of expression levels. On the other hand, random changes in the methylome are inducible by inhibiting the activity of certain epigenetic modifiers, for example, by applying small chemicals such as 5′‐azacytidine or 2′‐deoxy‐5‐azacytidine (Christman, 2002; Issa & Kantarjian, 2009). Interestingly, many of these chemical substances interfere with DNMT enzymes. As an example, the inhibition of DNMT1, required for maintenance of DNA methylation marks (Jin & Robertson, 2013) leads to dilution of DNA methylation in a dividing cell population. This has been used before to induce a hybridoma cell line to express natively silenced genes required for cholesterol production (Seth, Ozturk, & Hu, 2006). As such random changes in the methylation pattern may target other chromatin areas including regulatory sequences, such as enhancer or silencer regions (Feichtinger et al., 2016), these may well cause random changes in gene expression patterns. Such approaches would thus potentially enable the generation of gene expression profiles that were not present in the population before. Their effect on cell performance may be as likely detrimental as beneficial as is to be expected from any random approach. Feichtinger et al. (2016) showed that the DNA methylation pattern in CHO cells changed during adaptation to new culture conditions or when cells were sorted for high productivity but remained fairly constant as long as these new culture conditions were maintained. Histone modifications on the other hand correlate to gene expression during rapid environmental changes, as observed in standard batch or fed‐batch experiments. Thus histone modifications are a rapidly reversible response mechanism, while DNA methylation serves as the cell's inheritance for long‐term adaptation (Feichtinger et al., 2016; Hernandez et al., 2019).

Interestingly, established methods of recombinant protein production in mammalian cells already utilize substances affecting epigenetic control, such as the HDAC inhibitors valproic acid and sodium butyrate (NaBu) (Backliwal et al., 2008; Jiang & Sharfstein, 2008), or the stillbenoid resveratrol, that also affects epigenetic mechanisms (Fernandes et al., 2017; Toronjo‐Urquiza, James, Nagy, & Falconer, 2019). These substances are sometimes added during production processes to increase specific productivities (Toronjo‐Urquiza et al., 2019). Moreover, one commonly used chemical to achieve selection in CHO is methotrexate (MTX). MTX interacts and influences the formation of the cosubstrate molecule S‐Adenosyl methionine required for methylation of cytosines (Forster, McDonnell, Theobald, & McKay, 2017). This suggests that potentially the actual success of this selection system was enhanced by the combination of gene amplification and epigenetic changes.

**Figure 1:**
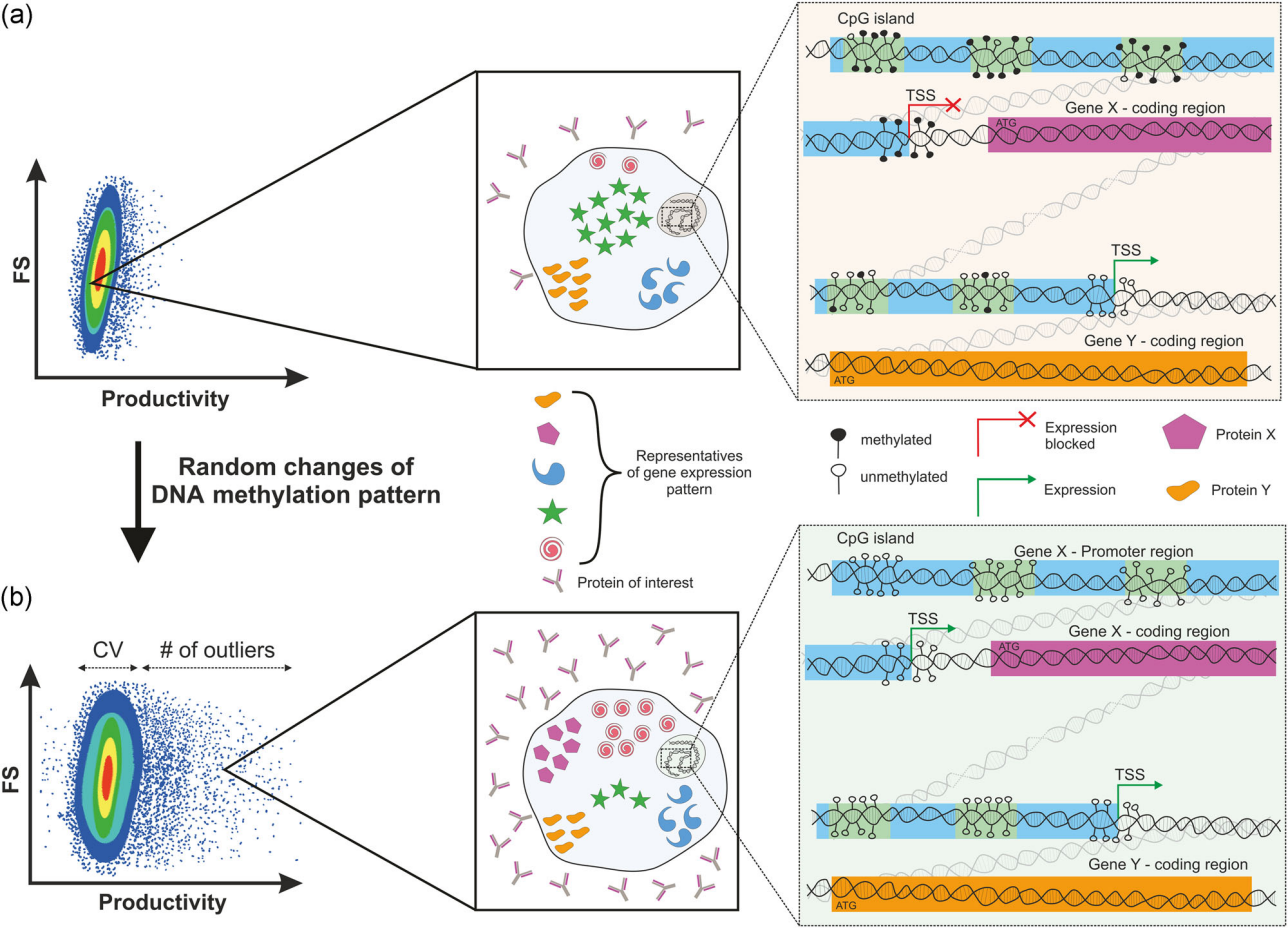
Changing process relevant phenotypes of Chinese hamster ovary (CHO) cells by epigenome manipulation. (a) Producing cell population before induced changes in DNA methylation pattern. First zoomed‐in square shows an example CHO cell that expresses a hypothetical set of genes. Zoomed‐in square on the right side indicates that, for example, gene Y is expressed due to mostly non‐methylated promoter region and transcription start site (TSS), whereas another gene X is not expressed due to predominantly methylated CpG islands in the promoter region and around the TSS. (b) Cell population after DNA methylation pattern change shows increased CV and higher numbers of outliers with increased productivity to the right of the main population, but also more low or nonproducing cells to the left. First zoom‐in square indicates a CHO cell that produces higher amounts of the recombinant product because its gene expression pattern changed (shown by different genes and expression levels inside the cell). Presumably, these changes in expression patterns were induced by demethylation of previously methylated CpG islands (as indicated in the right zoom‐in box) or by changes in methylation of regulatory regions [Color figure can be viewed at wileyonlinelibrary.com]

The present study investigates the systematic use of induced random changes in the DNA methylation pattern to generate long‐term alterations and higher diversity in cellular behavior within a population (Figure 1). For this purpose, the expressed DNA methylation modulating enzymes were transiently knocked down by small interfering RNAs (siRNAs) in CHO cells. Importantly, we intended to only transiently downregulate the expression of these enzymes to facilitate epigenome alterations within a defined time frame. A complete knock‐out, i.e. by CRISPR/Cas9, would result in cells that continue to constantly modulate their epigenome and thus would not be able not maintain potentially favorable DNA methylation patterns that had been isolated. Therefore, siRNAs against DNMT1 and 3a as well as the TET enzymes TET2 and 3 were repeatedly introduced to reduce their expression over a time period of 16 days. In fact, whole‐genome bisulfite sequencing (WGBS) revealed that the transient knockdown of DNMT1 and 3a reduces the total cytosine methylation from ∼75% to ∼38% in the treated samples and diversifies the pattern of DNA methylation across the population, suggesting that changes in the epigenome occurred. The same approach was applied to stable CHO production cell lines, allowing us to increase phenotypic diversity in a given cell population and to isolate cell pools with up to 1.5‐fold increased titers and specific production rates (qP) by sorting the outliers according to single‐cell secretion rate. These results provide proof‐of‐principle that (random) epigenetic modulation impacts and diversifies the observed cellular production phenotype. This also implies that during the establishment of a production cell line, in addition to differences in gene copy number and GOI transcription as controlled by the specific vector construct or the chromatin state of the integration site, differences in productivities or other cellular traits potentially arise due to changes in the epigenome. In addition, the presented strategy can be a supportive tool during the establishment of production cell lines, and a deeper understanding of the underlying mechanisms of epigenetic regulation may lead to novel approaches to enhance phenotypic stability in established cell lines.

## 2 MATERIALS AND METHODS

### 2.1 Cell culture

CHO‐K1 cells (ECACC 85051005) adapted to growth in suspension were routinely cultivated in CD‐CHO medium (Thermo Fisher Scientific, Waltham, MA) supplemented with 8 mM l‐glutamine (Thermo Fisher Scientific) and anticlumping agent (1:500 diluted, Thermo Fisher Scientific), incubated at 37°C, 7% CO_2_, humidified air, and 220 rpm shaking. Cells were passaged every 3–4 days by dilution to a cell density of 2 × 10^5^ cells/ml into 10 ml fresh medium in a 50 ml TPP® TubeSpin bioreactor (Techno Plastic Products AG, Trasadingen, Switzerland). CHO‐K1 Hy cell lines HyEpo A and HyEpo B were generated by random integration of a human erythropoietin–fragment crystallizable fusion protein (EPO–FC) fusion gene into CHO‐K1 Hy cells (Cytiva, Uppsala, Sweden), selection with blasticidin (InvivoGen, ‎San Diego, CA) at a concentration of 10 ng/µl and a limiting dilution subcloning step. During the subcloning step, InstiGRO™ CHO (SAL Scientific, Fordingbridge, UK) was added to the medium according to the manufacturer's instructions to support the outgrowth of single‐cell colonies. After isolation of the clones HyEpo A and HyEpo B, they were routinely cultivated as described for CHO‐K1, except that cells were kept in CD‐CHO medium (Thermo Fisher Scientific) supplemented with 6 mM GlutaMAX™ (Thermo Fisher Scientific) and 10 ng/µl blasticidin. CHO‐K1 Hy HyHer trastuzumab producing cells were received from Cytiva. They were routinely cultivated in CD‐CHO medium (Thermo Fisher Scientific) supplemented with 6 mM GlutaMAX™ (Thermo Fisher Scientific) and 75 µM MSX (Thermo Fisher Scientific).

### 2.2 Transfection

Transfection of siRNAs was performed as described earlier (Klanert et al., 2019). Briefly, siRNAs (Eurofins, Luxembourg City, Luxembourg) were transfected using the Neon® transfection system (Thermo Fisher Scientific) with the Neon® transfection system 100 µl kit (Thermo Fisher Scientific) according to the manufacturer's protocol. Therefore, 5 × 10^6^ cells were centrifuged at 170 rcf for 8 min and then resuspended in 100 µl buffer R. After the addition of 300 pmol of siRNAs, cells were transfected by applying one pulse with 1,700 V and 20 ms. A *mock* transfection and a nontargeting “scrambled” siRNA (AllStars Negative Control siRNA; Qiagen, Venlo, The Netherlands) were included as controls. Cells were allowed to recover in 10 ml of the respective media in a 50‐ml TPP® Tubespin bioreactors for 1.5−2 hr post‐transfection without shaking at 37°C, humidified air and 7% CO_2_. Subsequently, cultures were shaken at 220 rpm. For the repeated transfections of cells with siRNAs, this procedure was repeated every 4th day. All siRNA sequences are provided in Table S2.

### 2.3 Whole‐genome bisulfite sequencing

CHO‐K1 genomic (g)DNA was isolated on Day 2 after the 4th siRNA transfection (see above) using the DNeasy® Blood & Tissue Kit (Qiagen) according to the manufacturer's protocol. DNA was prepared using the NEBNext® Enzymatic Methyl‐seq Kit (New England BioLabs, Ipswich, MA) and analyzed by Illumina NovaSeq SP PE150. Raw reads were processed using Trim‐galore 0.6.0 (Martin, 2011) with a quality cutoff of 28 and trimming on all ends of 5 bp. Processed reads were aligned paired‐end mode to the Chinese hamster genome (Rupp et al., 2018) using the Bismark v0.22.1 pipeline (nondefault parameters: *N* = 1, score_min = L,0,−0.6; Krueger & Andrews, 2011). Bismark was also used to remove duplicate reads and to generate methylation profiles with default settings. Differential analysis was performed using DSS‐single (Wu et al., 2015). DSS settings comprised a smoothing span of 500 bp with a minimum differentially methylated region (DMR) length of 50 bp with ≥4 CpGs and *p* < .05 (Wald test). Raw methylation data were acquired using the R‐package bsseq (Hansen, Langmead, & Irizarry, 2012). Sequencing data is available under PRJEB37047.

Chromatin enrichment analysis was performed using data acquired from (Rupp et al., 2018), visible on http://cgr-referencegenome.boku.ac.at/ using the line “TP_4 (53 hr).” DMRs were assigned to the chromatin enrichment using the intersect command of the R‐package *Granges* (Lawrence et al., 2013). To test for enrichment, the following ratio was used

$$Chromatin\;Enrichment=\frac{\sum l_{chromDMR}}{\sum l_{DMr}}/\frac{\sum l_{chromGenome}}{\sum l_{Genome}},$$

where *l*_chromDMR_ is the length of intersections of each reported chromatin states with identified DMRs, *l*_DMR_ is the length of all DMRs identified, *l*_chromGenome_ is the length of each reported chromatin states identified in the reference genome, and *l*_Genome_ is the length of the whole reference genome.

### 2.4 Quantitative real‐time polymerase chain reaction

A number of 1 × 10^6^ cells were resuspended in 300 µl TRI reagent® (Merck KGaA, Darmstadt, Germany) and stored at −80°C until further purified. Total RNA was isolated using the Direct‐zol RNA Miniprep Kit (Zymo Research, Irvine, CA) according to the manufacturer's instructions. RNA concentration and quality were determined with a NanoDrop™ One (Thermo Fisher Scientific). Next, complementary DNA (cDNA) was generated from a total of 800 ng isolated RNA with the High‐Capacity cDNA Reverse Transcription Kit (Thermo Fisher Scientific) and RNase inhibitor (20 U/L; Thermo Fisher Scientific) according to the manufacturer's instructions. The cDNA samples were diluted 1:4 in nuclease‐free water after the reverse transcription. To quantify gene expression levels, cDNA templates were measured in quadruplets on a Rotor‐Gene Q (Qiagen) using the SensiFAST™ SYBR® Hi‐ROX Kit (Bioline Reagents, London, UK) according to the manufacturer's instructions, only the assay volume was downscaled to 10 µl, respectively. All primers for quantifications can be found in Table S3. Cycling conditions were 95°C 2 min, 40 cycles of 95°C 15 s, 60°C 20 s, and 72°C 20 s, and a melting curve was recorded from 65 to 99°C 0.5°C/step at 2 s for each step. The $2^{-\Delta \Delta C_{t}}$ method (Livak and Schmittgen, 2001) was used to quantify relative gene expression levels against one (glyceraldehyde 3‐phosphate dehydrogenase [GAPDH]), or three different reference genes (GAPDH, metabolism of cobalamin associated D; Brown, Gibson, Hatton, & James, 2018; and *cgriseus1B003354*; Hernandez et al., 2019) for more reliable results. Fold changes were calculated in relation to the *mock* sample. Normalization against three housekeeping genes should minimize the variation of expression of each single housekeeping gene.

### 2.5 Fluorescent activated cell sorting

Surface staining of EPO–FC or Immunoglobulin G (IgG)‐producing CHO‐K1 Hy cells was performed as described by Pichler et al. (2009) with minor changes. Briefly, 1 × 10^7^ per sample were centrifuged at 200 rcf for 8 min at 4°C and washed two times with cold HyClone™ Dulbecco's phosphate‐buffered saline (PBS; Cytiva, ‎Uppsala, Sweden). Next, the cells were stained in cold 200 µl staining solution (0.5% polyvinylpyrrolidone, 2 mM EDTA in PBS) containing 1:20 diluted F(ab')2‐goat anti‐human IgG FC R‐phycoerythrin (PE) conjugate (#H10104, Thermo Fisher Scientific) in the dark at 4°C for 30 min. The cells were washed one more time with cold PBS and then resuspended in 1 ml cold medium (as described above) containing 1:100 4′,6‐diamidin‐2‐phenylindol (DAPI). Fluorescent activated cell sorting was performed with a MoFlo® Astrios™ (Beckman Coulter, Brea, CA) cell sorter. Forward (FSC) and side scatter (SSC) was determined with a 488‐nm laser. PE fluorescence was detected with a 561 laser and a 579/16 bandpass (BP) filter. A violet 405‐nm laser was used for DAPI and the signal recorded in a 448/59 BP filter. The cells were always live gated based on FSC and SSC and a SSC area to height gate to identify single cells. An additional live‐gate was set on DAPI negative cells. Finally, 5,000 cells of the top 1% producing cells (PE signal) from each sample were sorted into 200 µl fresh selection medium containing penicillin‐streptomycin (10.000 U/ml penicillin, 10 mg/ml streptomycin; VWR Chemicals, ‎Radnor, PA) in a 96‐well plate (Greiner Bio‐One, Kremsmünster, Austria). Each sample was sorted in replicate pools. The sorted cell pools were incubated at 37°C, 7% CO_2_, humidified air, and static conditions for 11 days, then transferred to a 48‐well suspension plate (Greiner Bio‐One) and incubated at 37°C, 7% CO_2_, humidified air, 300 rpm shaking to allow further outgrowth. When ready, the cells were transferred to 50 ml TPP® TubeSpin bioreactor and cultivated as described above.

### 2.6 Batch culture

The respective cell lines were seeded at a cell density of 0.2 × 10^6^ cells/ml in 20 ml of the required medium (as described above) in a 50 ml TPP® TubeSpin bioreactor and incubated at 220 rpm shaking, 37°C, 7% CO_2_, and humidified air. Viable cell density (VCD) and viability were measured daily with the Vi‐Cell XR (Beckman Coulter, Inc., Brea, CA) based on trypan blue staining. In addition, supernatant samples were collected daily by centrifugation of 400 µl cell suspension at 200 rcf for 5 min and transfer of the supernatant to a new 1.5‐ml tube (Sarstedt AG & Co., KG, Nümbrecht, Germany). RNA samples from Day 5 after batch start were collected as described before.

### 2.7 Titer determination

Concentrations of the recombinant products were determined by bilayer interferometry measurements with the Octet® QKe (Pall Corporation, Port Washington, NY), equipped with Dip and Read™ Protein A Biosensors (Pall Corporation) according to the manufacturer's instructions. EPO–FC culture supernatants were either measured undiluted or diluted 1:2 in PBS + 0.1% Tween (pH 7.3). Trastuzumab supernatants were diluted 1:2, 1:4, or 1:8 depending on the time point of sampling. Serial dilutions (10 × 1:2 dilutions) of trastuzumab (BioVision, Milpitas, CA; starting with 100 µg/ml), were included as standards for absolute quantifications. EPO–FC and trastuzumab concentrations were quantified relative to a trastuzumab standard.

### 2.8 Evaluation of phenotypic changes

Determination of growth or productivity‐related phenotype changes was performed as described recently (Klanert et al., 2019). Briefly, the statistical software R version 3.6.0 (R Core Team. 2019) and the R‐package vicellR version 0.1.9 (Klanert et al., 2019) was used to calculate growth rates *µ* and specific productivities qP. All plots were generated using the R‐package ggplot2 (Wickham, 2016, p. 2).

## 3 RESULTS

### 3.1 Repeated knockdown of DNMTs generates genome methylation diversity

**Figure 2:**
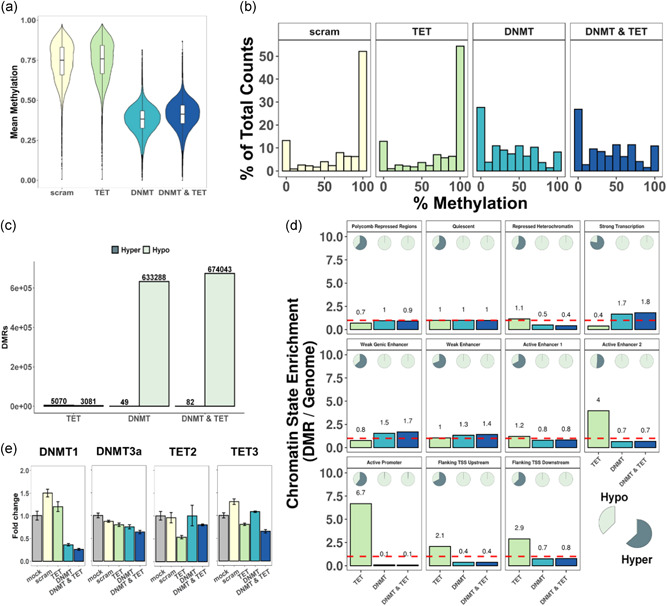
DNA methylation changes in CHO‐K1 cells. (a) Violin plot of mean methylation of CpGs in 1,000‐bp bins. (b) Counts of individual CpGs with defined percent of methylation across all reads. (c) Number of identified hyper‐ and hypomethylated regions (DMRs) compared to the *scram* control. (d) Chromatin state enrichment based on DMRs identified (shown in c). The red line indicates 1 (representing no change in enrichment). The circles display the share of associated hyper‐ or hypomethylated DMRs. (e) qPCR verification of siRNA efficacy‐based relative to the *mock* control. Quantified mRNA targets indicated above each plot. Error bars show the 95% confidence interval. CHO, Chinese hamster ovary; DMR, differently methylated region; DNMT, DNA methyltransferases; mRNA, messenger RNA; scram, scramble siRNA; siRNA, small interfering RNA; TET, ten‐eleven translocation; qPCR, quantitative polymerase chain reaction [Color figure can be viewed at wileyonlinelibrary.com]

To induce random changes in the DNA methylation pattern small molecules, such as 5′‐Azacytidine, which are described to interfere with the DNA methylation machinery, were tested. In addition, we designed siRNAs that should specifically downregulate the expressed enzymes of the DNA methylation machinery. These epigenome modulators were prescreened in the trastuzumab producing CHO‐K1 Hy cell line and in a CD4 producing CHO‐K1 cell line and evaluated based on the change in the population coefficient of variation (CV) of either secreted antibody or surface‐expressed CD4 as analyzed by flow cytometry (a summary of all tested chemicals is shown in Table S1 with selected chemicals visible in Figure S1). After treatment with small molecules, an increased CV was observed for most chemicals tested, but results were inconsistent with low reproducibility between independent experiments. The latter may be due to off‐target effects, the toxicity of some of these chemicals or to the inherent randomness of the approach. Subsequent work, therefore, focused on the direct knockdown of enzymes that maintain, generate, or remove DNA methylation. Specifically, the DNMTs 1 (gene ID: 100762713) and 3a (gene ID: 100771064), as well as the TET enzymes 2 (gene ID: 100769811) and 3 (gene ID: 100769509) were targeted. DNMT3b and TET1 were excluded as they are not expressed in CHO cells (Hernandez et al., 2019; Singh, Kildegaard, & Andersen, 2018). Different siRNAs were screened individually and in combination for their efficiency by transfection of CHO‐K1 cells and subsequent quantitative polymerase chain reaction (qPCR) on different days post transfection (Figures S2 and S3). siRNA mixes were introduced into CHO‐K1 cells for four consecutive times every four days to evaluate the impact on DNA methylation. Cells transfected with solely water (“*mock*”) or with a scrambled, nontargeting siRNA (“*scram*”) were used as controls. Monitoring the VCD and viability over the course of knockdown did not show major differences between samples. Still, decreasing VCD values after each transfection indicate a general impact of multiple, successive transfections irrespective of whether they were control or which siRNA was used (Figure S4). The knockdowns of the target genes were confirmed by qPCR evaluation of expression levels 4 days after the first transfection and were shown to work efficiently for all but the DNMT3a target (Figure 2). After these multiple interferences, genomic DNA was isolated and analyzed by WGBS. The results clearly show that in the samples where DNMT1 and 3a had been knocked down, total DNA methylation of cytosine was reduced from a median of 0.750 (scrambled siRNA control) to 0.382. This reduction was also found in samples were both DNMTs and TETs were knocked down (median = 0.413). In contrast, in samples in which only TET 2 and 3 were knocked down no obvious change of the methylation content or distribution (median = 0.759) was observed (Figure 2a). Moreover, in the samples treated with scrambled or anti‐TET siRNA, the majority of CpGs were completely methylated, whereas in the samples treated with anti‐DNMT siRNA the majority was completely demethylated (Figure 2b) and an increased number of CpGs were partially methylated, indicating a high variation of methylation state in individual cells within the population. In comparison to the *scram* sample, the DNMT knockdown samples show a total of 633,337 or 674,125 DMRs, whereas the TET knockdown shows only 8,151 (Figure 2c). Next, we identified DMR enrichments within certain genomic contexts. Therefore, the genome was categorized into specific chromatin states based on the presence of specific histone tail modifications as described in Feichtinger et al. (2016) and Rupp et al. (2018). The amount of methylated cytosines in all of the respective chromatin regions was substantially lowered upon knockdown of DNMT (Figure S7). Subsequently, the regions were screened for the enrichment of these DMRs (Figures 2d and S8). Interestingly, upon knockdown of the DNMTs, enriched numbers of DMRs, according to the relative length of these chromatin states, were found in regions categorized as strong transcription, weak genic enhancer, and weak enhancer (enriched by ∼1.3 to 1.8‐fold), while regions of active promoters or flanking active transcription start sites contained less DMRs than they should according to their length and assuming random distribution of DMRs across the genome. Strikingly, the enrichments in TET knockdown samples showed a completely different picture: strong enrichments of DMRs in active promoters, transcription start sites, or active enhancers, but depletion in loci of strong transcription and weak enhancers. Associated DMRs indicated a constant pattern: the massive demethylation generated by a DNMT knockdown lead to almost solely hypomethylated DMRs in all chromatin states, whereas the knockdown of TET enzymes lead mainly to hypermethylated DMRs, but with a considerable share of hypomethylated regions in the analyzed chromatin states. Quantification of siRNA target mRNA levels revealed that the siRNAs worked specifically and reduced the respective target expression levels (Figure 2e). In summary, these results highlight the success of an siRNA‐induced change in the DNA methylation pattern by a reduction of DNMT1 and 3a expression.

### 3.2 Induced epigenetic modulation diversifies cell behavior in low‐producing CHO cells and allows isolation of cells with increased productivity

**Figure 3:**
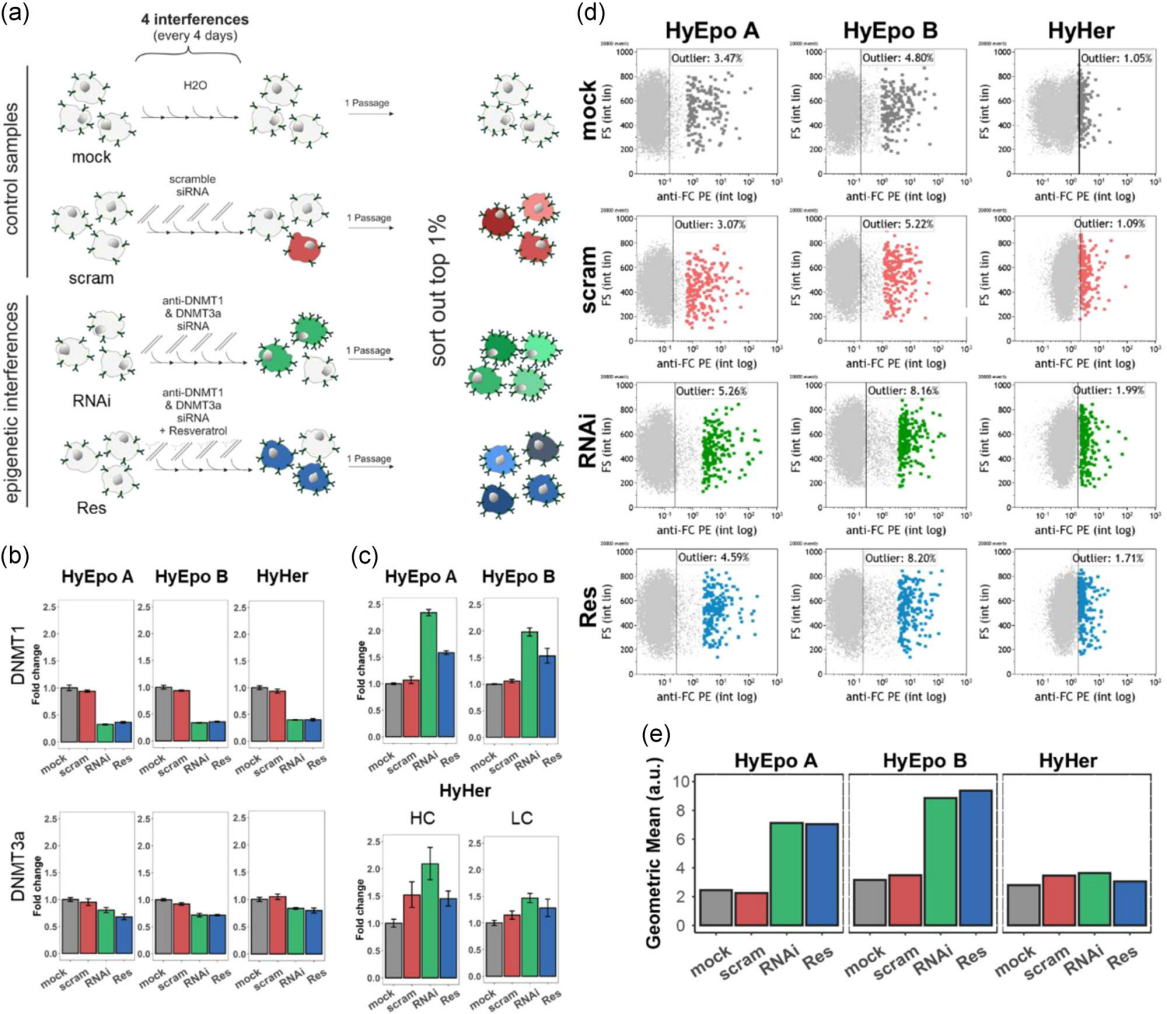
Epigenetic modulation of CHO‐K1 Hy producer cell lines. (a) Schematic overview of the workflow and sample description. (b,c) qPCR verification of DNMT knockdown (b) and recombinant mRNA expression (c) performed from the pools after the third transfection. Quantified mRNA targets indicated above each plot. Error bars show the 95% confidence interval. (d) Flow cytometry plots at the time point of sorting. Live cells were stained for secreted product with an anti‐human FC PE conjugate at 4°C. FS, forward scatter. Columns show the individual cell lines. Rows show the samples as indicated in (a). “% outlier” shows the percentage of cells in the gate set outside the border of the main population. Colored, bigger dots show the top 1% cells that were sorted. (e) Geometric mean values of top 1% as shown in (d). CHO, Chinese hamster ovary; DNMT, DNA methyltransferases; FC, fragment crystallizable; mock H_2_0 control; mRNA, messenger RNA; Res, resveratrol; RNAi RNA interference; scram, scramble siRNA; siRNA, small interfering RNA [Color figure can be viewed at wileyonlinelibrary.com]

The approach of modulating the DNA methylation pattern was applied to recombinant CHO‐K1 Hy cells stably producing a human EPO–FC fusion protein expressed from a CMV promoter and enhancer. In addition to the anti‐DNMT1 and 3a siRNA treatment alone (sample called *RNAi*), a sample of cells was treated with these siRNAs and in addition incubated in the presence of resveratrol (sample *Res*). Resveratrol is a stilbenoid (naturally occurring phenol), that was described to be beneficial for human health, mostly due to its antioxidant properties (Zhu et al., 2012), but that also has multiple connections to interfere with the epigenome (Fernandes et al., 2017; Toronjo‐Urquiza et al., 2019; Zhu et al., 2012). Hence, this compound was added to see whether a different, potentially more pronounced effect is achievable by the combination of two different epigenetic modulation strategies. The clones HyEpo A and HyEpo B produced the fusion protein EPO–FC at low specific production rates (qP < 1 pg/[c × d]). These clones had been subcloned after blasticidin selection, but neither been subjected to amplification nor sorted for high productivity. After successive knockdowns of DNMT1 and 3a expression (and addition of resveratrol in the Res sample), an increase in CV by ∼1.3 ‐ 1.45‐fold was observed over the control samples when live cells were stained for secreted EPO–FC and analyzed in flow cytometry (Figure S5). As a consequence, the number of outliers was increased. To verify these findings and subsequently isolate cells with improved production capacities, the experiment was repeated. In addition, a third cell line, CHO‐K1 HyHer that produced the monoclonal IgG antibody trastuzumab (market name: Herceptin®) at high levels (qP ∼20 pg/[c × d]), was treated in the same way to evaluate the effects of such epigenetic changes on a cell line already producing at industrial‐relevant levels. This cell line had been selected by the glutamine‐synthetase (GS) and methionine sulfoximine (MSX) system. Both, heavy and light chain (HC or LC) were expressed under control of an EF1α promoter. As described before, the siRNAs were applied four consecutive times every four days (Figure 3a). A *mock* (cells transfected with water) and a *scram* (cells transfected with a scrambled siRNA) sample were included as controls. After treatment, cells were passaged once to allow full recovery after transfections, then stained for secreted product and the top 1% highest producing cells were sorted into pools of 5,000 cells/well (Figure 3a and d). Following VCD and viability over the transfection period showed again a general impact of multiple transfections on cellular growth. In addition, we observed a more profound impact of the DNMT knockdowns on viability and also VCD (Figure S6) in the samples HyEpo A and B, but to a lesser extent in HyHer. After the knockdown period, population viability and VCD recovered again quickly. At the point of sorting, the percentage of outliers was increased substantially in the *RNAi* and *Res* samples of HyEpo A and HyEpo B, whereas no significant change was observed in the HyHer cells (Figure 3d). Moreover, the geometric mean of the top 1% HyEpo A and HyEpo B *RNAi* and *Res* was increased by ∼3‐fold, again with no significant change for HyHer cells (Figure 3e). qPCR verification of target knockdown in the pools before sorting showed that the siRNAs were effective (Figure 3b). Quantification of the recombinant mRNA, either for EPO–FC or the HC and LC of trastuzumab, respectively, indicated that their transcription was increased upon knockdown of DNMT1 and 3a (Figure 3c). This effect is seen more prominently in the case of EPO–FC expression, in particular for clone HyEpo A.

**Figure 4:**
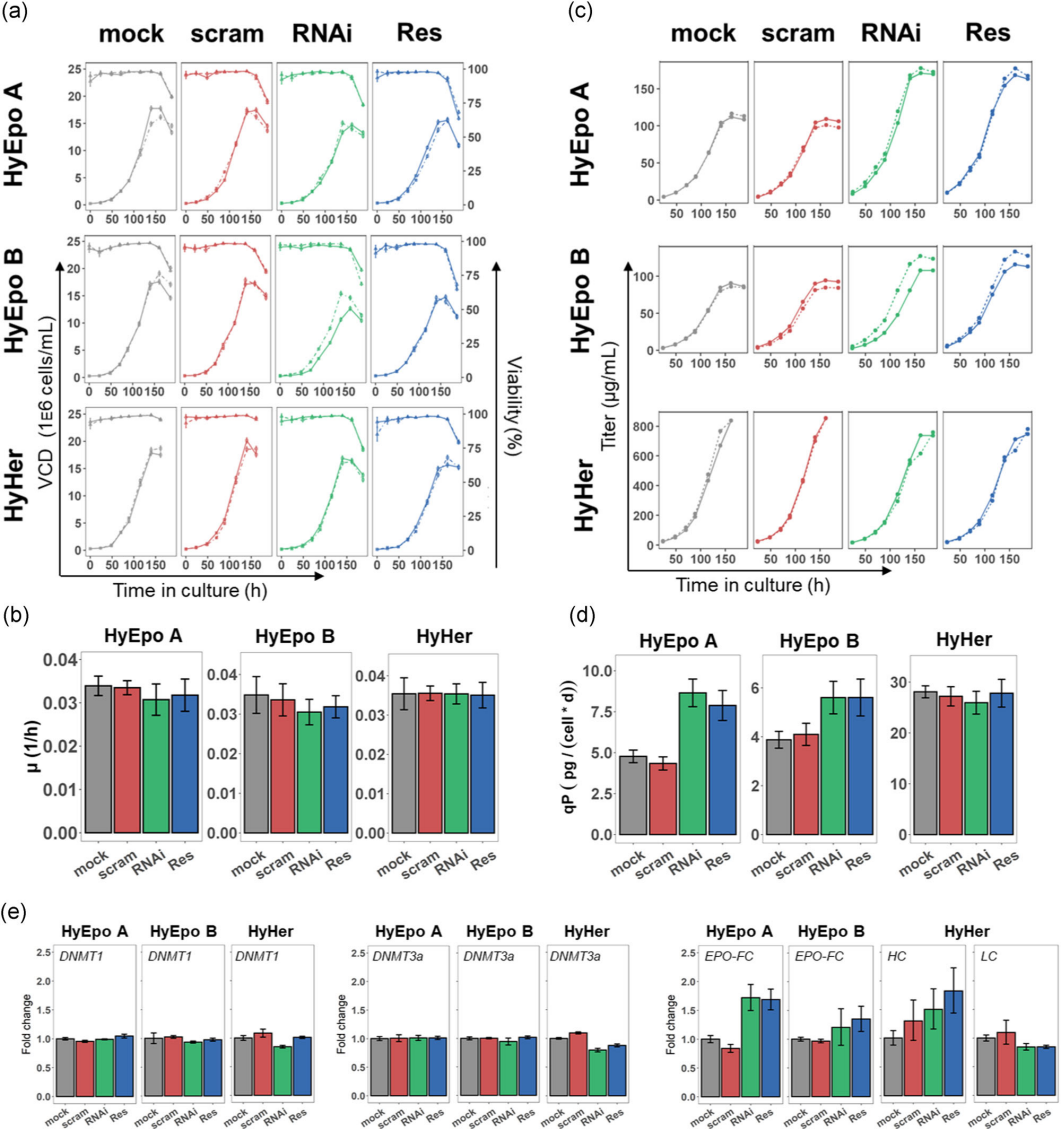
Evaluation of phenotypic characteristics in the sorted CHO‐K1 Hy producer cell lines. (a) Viable cell densities (VCD; lower lines) and viabilities (upper lines) over the time of culturing. Full and dotted lines represent individual biological replicates (n = 2). (b) Average growth rates *µ*. Error bars show the 95% confidence interval. (c) Recombinant titer of the respective products quantified by Octet® measurements. Lines as in (a). (d) Average specific production rates (qP). Error bars show the 95% confidence interval. (e) qPCR verification of DNMT expression and recombinant mRNA expression. Quantified mRNA targets indicated above each plot. Error bars show the 95% confidence interval. CHO, Chinese hamster ovary; DNMT, DNA methyltransferases; EPO–FC, erythropoietin–fragment crystallizable fusion protein; mock H_2_0 control; mRNA, messenger RNA; Res, resveratrol; RNAi RNA interference; scram, scramble siRNA; qPCR, quantitative polymerase chain reaction [Color figure can be viewed at wileyonlinelibrary.com]

Next, the sorted cell pools were evaluated in a small‐scale batch experiment in shake flasks to characterize changes in production and/or growth behavior. There were no significant changes in growth, expect that the *RNAi* and *Res* samples from all three cell lines grew to slightly lower maximum VCDs (Figure 4a). However, the growth rate was similar in all samples (Figure 4b). The final titer in the HyEpo A producing cell line was increased from ∼105 µg/ml (similar in *mock* and *scram* samples) to ∼165 µg/ml in both the *RNAi* and the *Res*‐treated samples (Figure 4c), corresponding to an increase in both titer and qP by ∼1.5‐fold (Figure 4d). Similarly, but to a different extent, the titer in the HyEpo B cell line increased from ∼90 µg/ml (*mock* and scram) to ∼130 µg/ml in the *RNAi* and *Res* samples (Figure 4c), corresponding to an increase of qP by 1.4‐fold (Figure 4d). In the IgG‐producing HyHer sample no significant differences were observed (Figure 4d). The expression of DNMTs was found to be restored to similar levels as in the controls. Interestingly, for EPO–FC we could still detect increased transcript levels in the case of HyEpo A, but not HyEpo B (Figure 4e).

## 4 DISCUSSION

In this study, we demonstrated that a targeted knockdown of DNMTs via RNAi is highly effective in randomly demethylating the genome in the individual cells of a population (Figure 2), leading to a diversification of the observed phenotype (i.e., an increase of the percentage of outliers as analyzed by flow cytometry). This diversification of the population enabled us to isolate cell pools with a 1.4–1.5‐fold higher productivity (both total titer and qP) compared to controls (Figures 3 and 4). Thus, the concept of intentionally inducing (random) changes in the DNA methylation pattern to isolate cells with outstanding properties was demonstrated to be successful.

The use of siRNAs against enzymes that control DNA methylation has the advantage of little off‐target effects and of maintaining the cells at high viability and growth. In contrast, small molecule chemicals that are often used to change DNA methylation in cancer research, such as 5′‐azacytidine, are in most cases cytotoxic, can additionally incorporate into RNA or interact with other proteins in the cell (Christman, 2002; Davidson, Crowther, Radley, & Woodcock, 1992; Issa & Kantarjian, 2009; Li, Olin, Buskirk, & Reineke, 1970; Palii, Emburgh, Sankpal, Brown, & Robertson, 2008). Consequently, such chemicals can strongly impact and distort the observed cellular phenotypes not only by epigenetic effects. In addition, as mentioned earlier, these small‐molecule chemicals are often highly toxic and mutagenic, therefore requiring special precautions when working with them, whereas siRNA usually do not represent any risk. Nevertheless, we also tried to add resveratrol in addition to the siRNA treatment, a rather safe chemical, but did not see an enhanced effect over administering only the *RNAi* (Figures 3 and 4), suggesting that the targeted knockdown of epigenetic enzymes is sufficient. While we observed only minor knockdown efficiencies of DNMT3a mRNAs levels (Figure 2e), potentially due to the already low expression level of this enzyme (Hong, Jiang, Kim, Li, & Lee, 2014), the knockdown of DNMT1 was efficient and is likely mainly responsible for the observed decrease in DNA methylation. DNMT1 is required for maintaining the existing DNA methylation, whereas DNMT3a and 3b (which is not expressed in CHO cells) generate de novo methylation of CpGs (Jin & Robertson, 2013). Consequently, upon knockdown of DNMT1, the existing methylation pattern in the cells cannot be maintained and is reduced with each cell division. On the other hand, a knockdown of DNMT3a will mainly prevent the generation of new methylation at previously unmethylated positions but will have little impact on the existing marks. While the low impact of TET knockdown was unexpected (Figure 2c), it is logical that the effects were mainly focused on chromatin states of the cellular genome which usually contain few methylated CpGs, such as active promoter regions. The knockdown of TETs thus predominantly results in hypermethylated DMRs. Upon the knockdown of DNMTs, DMRs were enriched in genomic regions associated with initially rather high CpG methylation such as actively transcribed regions or regulatory regions such as enhancers (Feichtinger et al., 2016). In both cases, however, there is an enrichment of DMRs in chromatin areas that are important for (efficient) gene expression and its regulation, rather than a completely random distribution across the chromatin states of the entire genome.

Applying the more effective knockdown strategy of DNMTs to producer cell lines leads to an increased number of outliers (i.e. higher producing cells) and increased geometric means (indicating higher production rates) in the top 1% of EPO–FC producing CHO‐K1 Hy cell lines. However, no obvious changes or further improvements were detected in the previously established high producing IgG CHO‐K1 HyHer cell line (Figure 3). There are several possible explanations for these different responses (or lack thereof): An admittedly simplistic one is that the application of siRNAs and the resulting interference with the cellular DNA methylation pattern is random. Therefore, the results may vary if applied multiple times. A more straightforward explanation is that the highest benefit of epigenetic modulation can be obtained when the overall transcriptome of a population is still far from optimal. The EPO–FC cell lines were at an early stage of development, where essentially only blasticidin selection and a single round of subcloning had been performed, and only a limited number of clones were screened (< 96). Adaptation time during selection was comparatively short, only around 2 weeks (since transfection). Consequently, these clones had quite low specific production rates in the beginning and did not have much time to adapt to the challenge of production. On the other hand, the IgG producer CHO‐K1 HyHer had run through a full CLD program and showed already high production rates, which are likely owed to efficient transcription of the GOI mRNA(s) as well as a favorable gene expression pattern capable of supporting high production rates of the GOI (Seth et al., 2006). Interfering with such an already optimized transcriptome pattern entails the danger of actually making things worse which is what we partly observed. While the transcription of the heavy chain was increased, there was no corresponding increase in LC transcription. Such an unbalanced expression of light and heavy chain has been described to be detrimental to efficient production before (Ho, Wang, Song, & Yang, 2015; Schlatter et al., 2005).

On the other side, during CLD, a diversification of the existing, suboptimal transcriptome pattern can generate the highest benefit, as the number of outliers with outstanding phenotypes is increased. These can be efficiently isolated by cell sorting and higher frequencies of such outstanding performers potentially speeds up the adaptation and optimization process. As shown in Figure 4, the increase in qP and the total titer achieved by this approach in the EPO–FC cell lines ranges between 1.4–1.5‐fold, without any of the standard CLD tools such as gene amplification.

Another effect observed in this case was the increase in transcription of the GOI itself. During random integration of a GOI into the host genome, the locus of integration is not defined so that the surrounding chromatin state has an impact on its expression, irrespective of the promoter used. As chromatin states are defined by their histone modification patterns which in turn interact with DNA methylation (Du, Johnson, Jacobsen, & Patel, 2015; Zhao et al., 2016), the random epigenetic modulation applied in this study could also result in subtle changes in the chromatin states surrounding the integration site, thus enhancing transcription. Others have used tools for targeted alterations of histone modifications or for attracting transcription factors to enhance gene expression (Karottki et al., 2020). However, these changes are transient and in effect only for as long as the corresponding “writer” is active and provided to cells. Changes in DNA methylation on the other hand are long‐lasting and are passed on to progeny cells, thus resulting in stable changes in phenotypes (Marx et al., 2018). In addition to the chromatin states, also the methylation status of the promoter itself can be important for gene expression levels (Osterlehner, Simmeth, & Göpfert, 2011). Intriguingly, a recent study demonstrated that upon a DNMT3a knock‐out also the type of transgene promoter impacts the expression levels (Jia et al., 2018). In line with this publication, we also found enhanced expression levels of the EPO–FC transgenes from the CMV promoter plus enhancer, but lower impacts on the HC and LC genes that were controlled by the EF1α promoter.

Overall, the here described results offer a proof‐of‐principle for the applicability and importance of epigenetic modulation to induce changes in phenotypes of mammalian cell factories. Intriguingly, the randomly induced changes allowed us to isolate cell pools with increased production capacities. In addition, these results indicate that epigenetic regulation plays an important, so far largely neglected role in the establishment of production cell lines and potentially explains the observed heterogeneity of subclones and cell lines, depending on their history and culture conditions (Pilbrough et al., 2009). While clonal instability so far typically refers to production instability, which was often linked to epigenetic changes, both DNA methylation and/or histone marks, in the respective promoter of the transgenes (Brooks et al., 2004; Moritz, Woltering, Becker, & Göpfert, 2016), here we use the term stability in a wider sense referring to the entire phenotype and behavior of a cell line rather than just productivity. In this larger sense, a deeper understanding of epigenetic mechanisms and their regulation in production cell lines under given cultivation conditions may also contribute to our knowledge on what is required to maintain a given favorable phenotype over prolonged periods and thus may ultimately lead to novel strategies to improve stability.

## CONFLICT OF INTERESTS

Andreas Jonsson, Daniel Ivansson, and Ann Lövgren are employees of Cytiva. The authors declare that there are no conflicts of interests.

## Supporting information

Supporting information
