# Supplementary material for: Random epigenetic modulation of CHO cells by repeated knockdown of DNA methyltransferases increases population diversity and enables sorting of cells with higher production capacities
Source: Biotechnol Bioeng. 2020 Jul 24;117(11):3435–47. doi: 10.1002/bit.27493 (PMC7818401; doi:10.1002/bit.27493)
Supplement: Supplementary file 1 — Supporting information [file BIT-117-3435-s001.pdf]

Supplementary table 1: Chemical substances related to epigenetics and the respecitive concentrations tested in CHO for their effect on the cellular phenotype.

Chemical	Tested concentration
5-Azacytidine	100 nM, 1 µM, 2 µM, 3µM, 4 µM, 5 µM, 10 µM, 15 µM
5-Aza-2′-deoxycytidine	100 nM, 1 µM, 3 µM, 5 µM, 10 µM, 15 µM
Zebularine	100 nM, 1 µM, 10 µM, 50 µM, 100 µM, 200 µM
Resveratrol	100 nM, 1 µM, 5 µM, 10 µM, 20 µM, 50 µM
Curcumin	100 nM, 1 µM, 5 µM, 10 µM, 20 µM, 50 µM
EGCG	100 nM, 1 µM, 5 µM, 10 µM, 50 µM, 100 µM, 200 µM
Genistein	100 nM, 1 µM, 5 µM, 10 µM, 20 µM, 50 µM
Methotrexate (MTX)	100 pM, 1 nM, 10 nM, 100nM, 200 nM, 400nM
Procanamid	100 nM, 1 µM, 10µM, 100 µM, 500 µM, 1 mM
RG108	100 nM, 1 µM, 5 µM, 10 µM, 50 µM, 100 µM, 200 µM

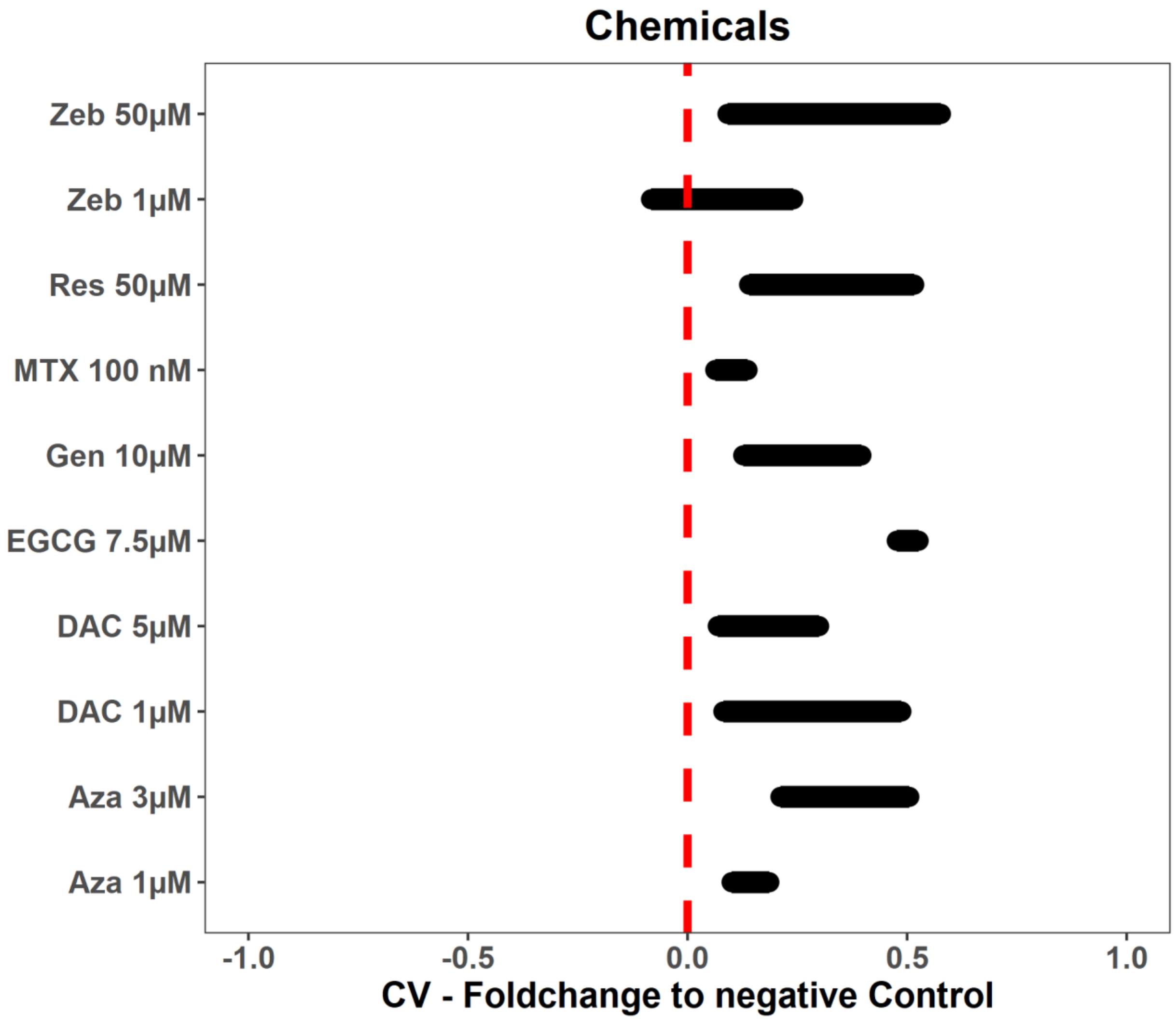

Supplementary figure 1: Effects of chemical substances in selected concentrations on the cellular population coefficient of variation (CV) of CHO-CD4 cells.

Supplementary table 2: siRNA sequences used to knock-down DNMT1, DNMT 3a, TET2 or TET3.

Name	Sequence (5' --> 3')
DNMT1-1	CUCACGCCAUCUAUGAAGA
DNMT1-2	CAGCUCGACUGUAUAUGAA
DNMT1-3	GCAAGGUCAAGGUUGUCUA
DNMT3a-1	GCAUCCACUGUGAAUGAUA
DNMT3a-2	GGACAUUUGUAUCUCAUGU
DNMT3a-3	CCAGGUCAAAUUCCAUAAA
TET2-1	GCUACUAAUGAGUUGUCUU
TET2-2	GCUCUGAGCAGUAUUUAAA
TET2-3	CCACAACCCGAAUAAAUAA
TET3-1	CCAACAUAACCUCUACAAU
TET3-2	CCUUCGGCUACUAUGGCUU
TET3-3	GGACCUGGCUACUGAAGUU

Supplementary table 3: Sequences of qPCR primers.

Name	Sequence (5' --> 3')
DNMT1_fwd	TTGAATCCCCTCCCAAGACC
DNMT1_rev	TCAAGTTGCTCCAGGACCTT
DNMT3a_fwd	TGCCAGAACTGTAAGAACTGC
DNMT3a_rev	GTAGCAGTTCCAGGGGTCTT
TET2_fwd	AGACTCAATATGATATCCCACCCT
TET2_rev	TCCATGATTGTTCTAATAGCTGCC
TET3_fwd	ATGTACTTCAACGGCTGCAAAT
TET3_rev	CTTGTACAGGGGAGCAACTTC
EPO-FC_fwd	CATGGGGGTGCACGAATGTC
EPO-FC_rev	CAAGCTGCAGTGTTCAGCAC
Traz_HC_fwd	GCTGAACGGCAAAGAGTACA
Traz_HC_rev	GTAGAAGCCCTTCACGAGAC
Traz_LC_fwd	GCTGAACAACTTCTACCCCC
Traz_LC_rev	CTTGTGCTTCTCGTAGTCGG
Gapdh_fwd	AACTTTGGCATTGTGGAAGG
Gapdh_rev	ACACGTTGGGGGTAGGAACA
mmadhc_fwd	TGTCACCTCAATGGGACTGC
mmadhc_rev	CAGGTGCATCACTACTCTGAAAC
cgriseus1B003354_fwd	GGAGATGGAGCTGTGGATGA
cgriseus1B003354_rev	AGAACTCGGTGGTTGGGAAT

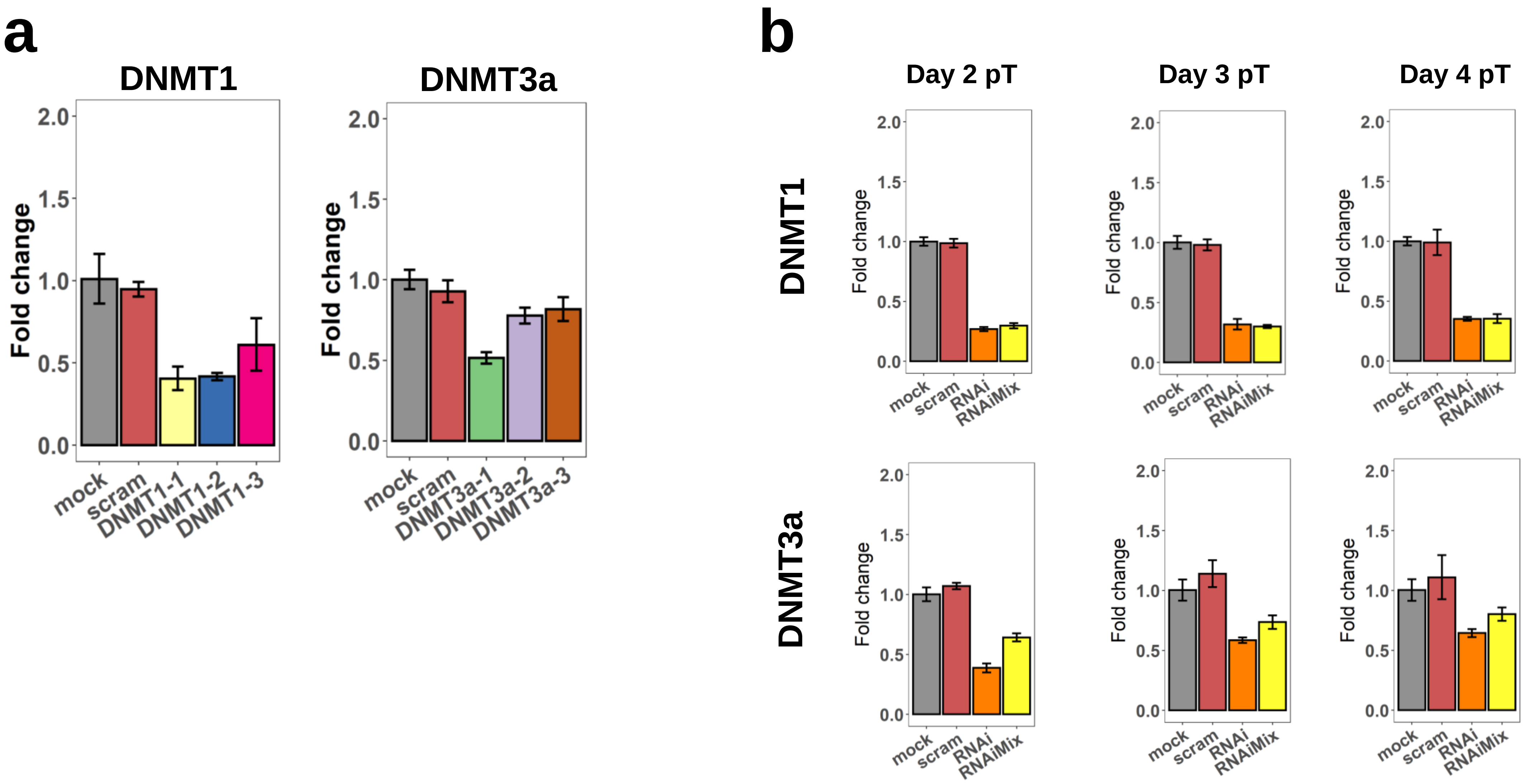

Supplementary figure 2: DNMT knock-down efficiency by RNAi.
(a) Quantified knock-down efficiencies of DNMT1 and 3a siRNAs 1-3 by qPCR on day 3 post transfection. Samples relative to mock sample (b) Following DNMT knock-down efficiencies over day 2-4 post transfection (pT). RNAi = either DNMT1 or DNMT3a siRNAs 1-3 ; RNAiMix = mix of all DNMT1 and 3a siRNAs. Samples relative to mock sample

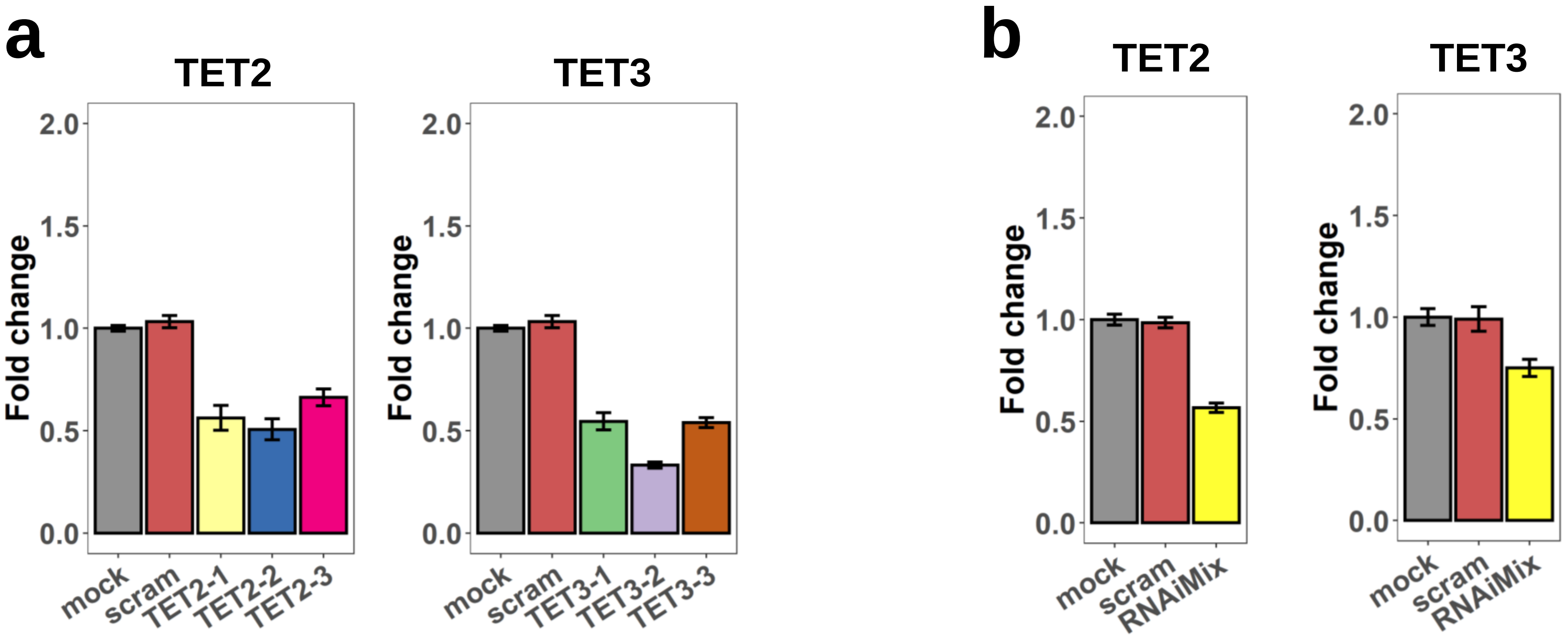

Supplementary figure 3: TET knock-down efficiency by RNAi.
(a) Quantified knock-down efficiencies of TET2 and 3 siRNAs 1-3 by qPCR on day 3 post transfection. Samples relative to mock sample (b) Combined TET knock-down efficiencies on day 3 post transfection. RNAiMix = mix of all TET2 and TET3 siRNAs. Samples relative to mock sample

a

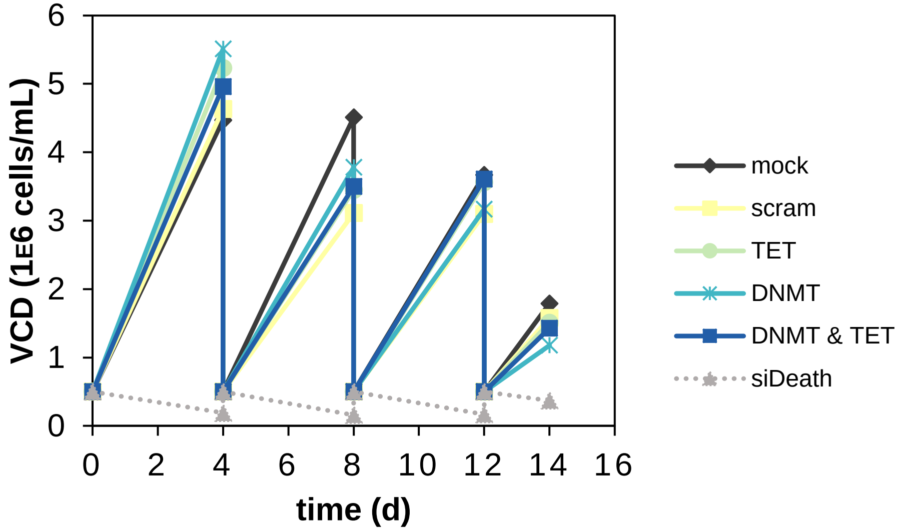

b

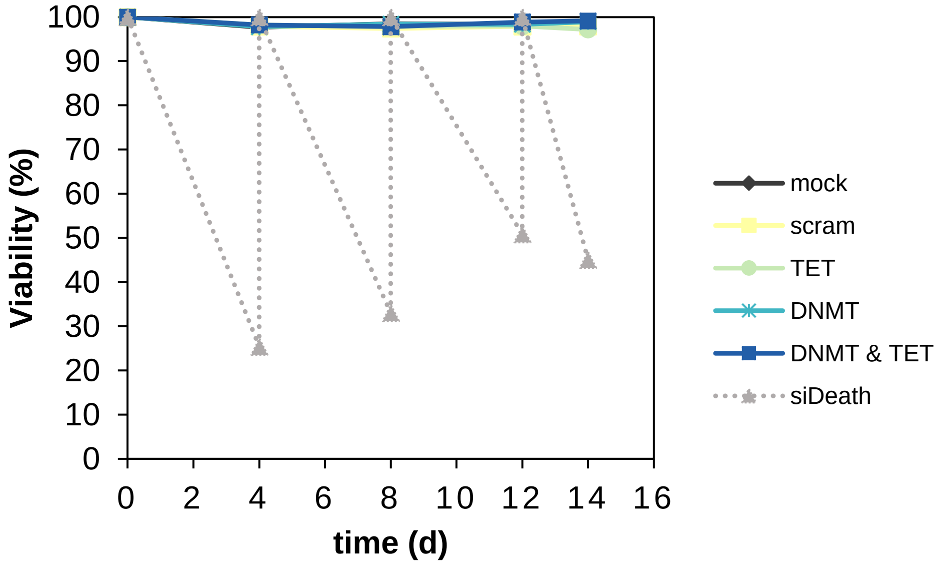

Supplementary figure 4: Growth trends of CHO-K1 transfected with siRNA mixes.
(a) Viable cell density (VCD) profile. The cells were re-transfected every four days, explaining the zic-zac shape of the curve. (b) Viability (%) of the transfected cells. CHO-K1 for siDeath control were exchanged every time of a new transfection, therefore the viability always jumps to assumed 100%.

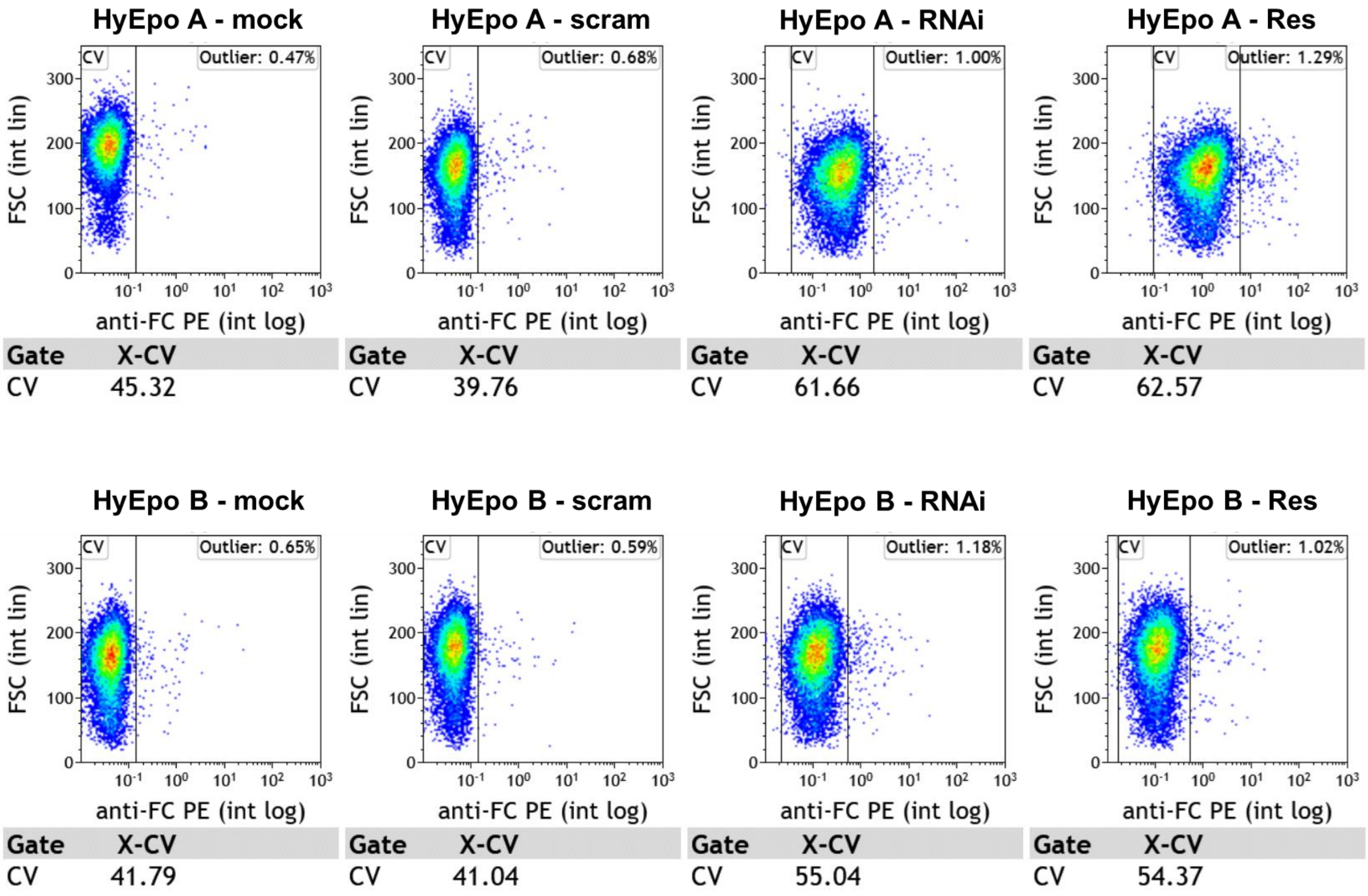

Supplementary figure 5: Flow cytometry plots of HyEpo A and B cells. Cells were stained with an anti-human FC F(ab) PE conjugate at 4°C. Statistics show calculated CV for the main population (gate “CV”). Gate “outlier” shows the % of gated cells.

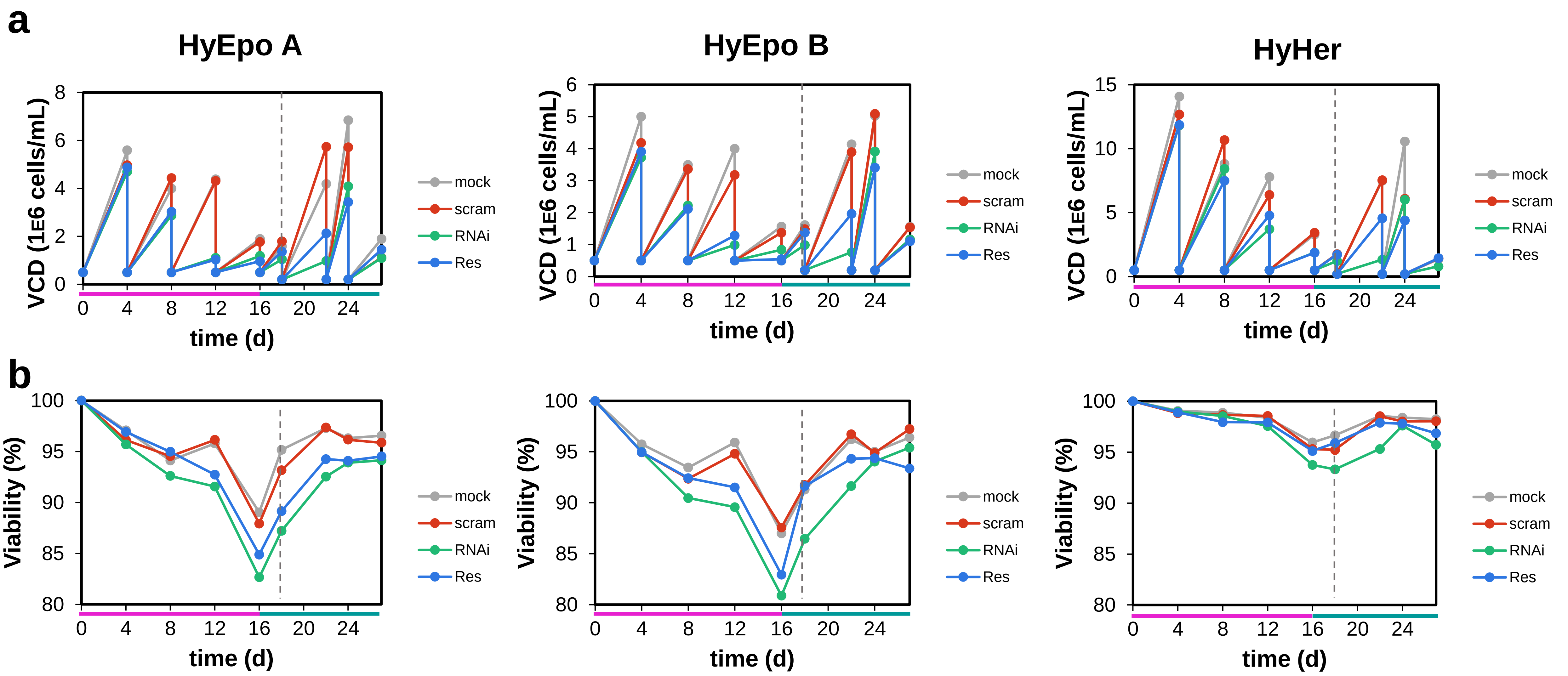

Supplementary figure 6: Growth trends of CHO-K1 HyEpo A, B and HyHer transfected with siRNA mixes.
(a) Viable cell density (VCD) profile. (b) Viability (%) of the transfected cells. Pink line indicates time of DNMT knock-down, and teal line indicate that in this period the cells were “normally” passaged. Dotted line shows timepoint of sorting the top 1%.

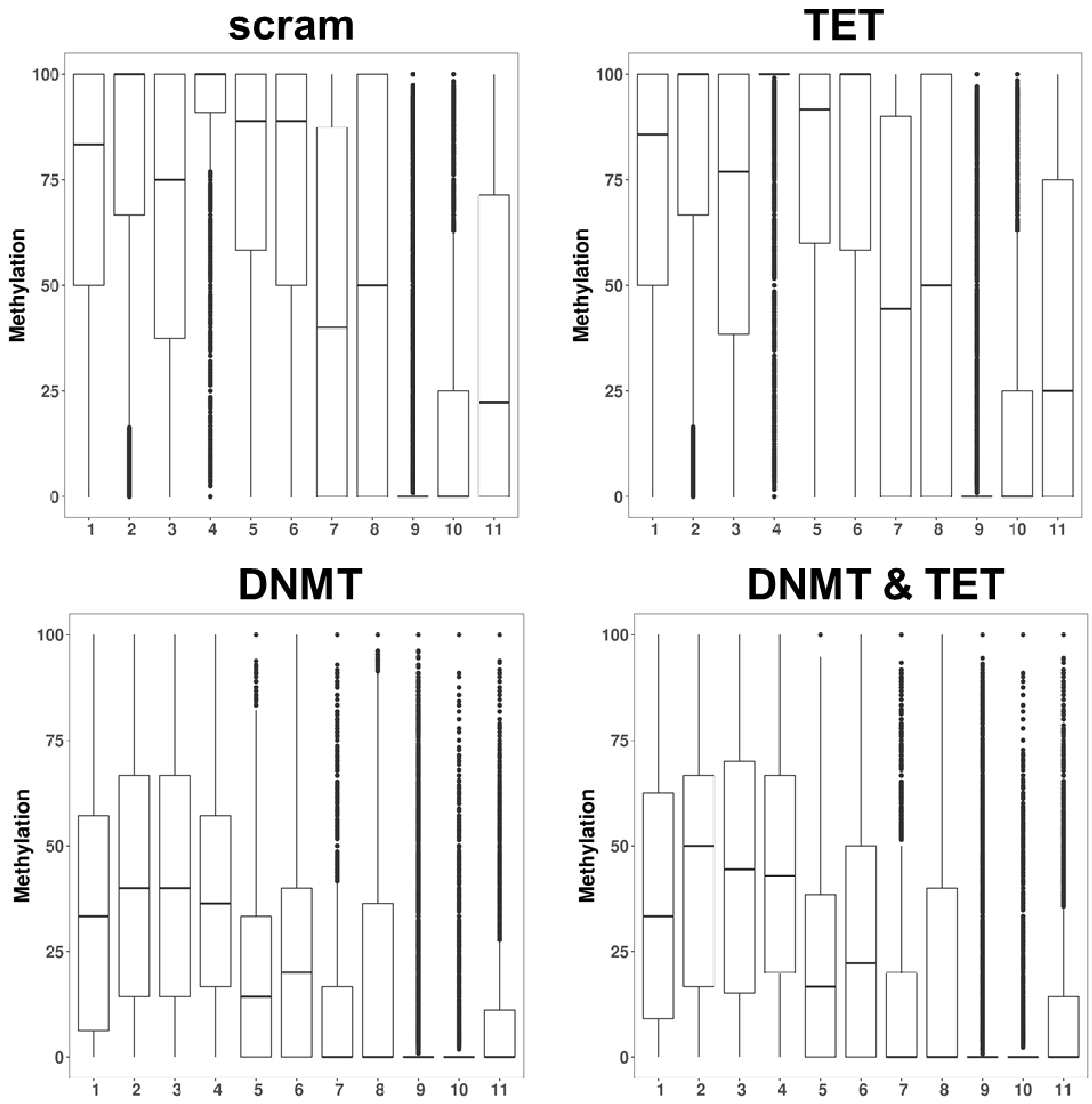

1 - Polycomb Repressed Regions

2 - Quiescent

3 – Repressed Heterochromatin

4 – Strong Transcription

5 – Weak Genic Enhancer

6 – Weak Enhancer

7 – Active Enhancer 1

8 – Active Enhancer 2

9 – Active Promoter

10 – Flanking TSS Upstream

11 – Flanking TSS Downstream

Supplementary figure 7: DNA methylation in defined chromatin regions. y-axis shows % methylated Cytosines.

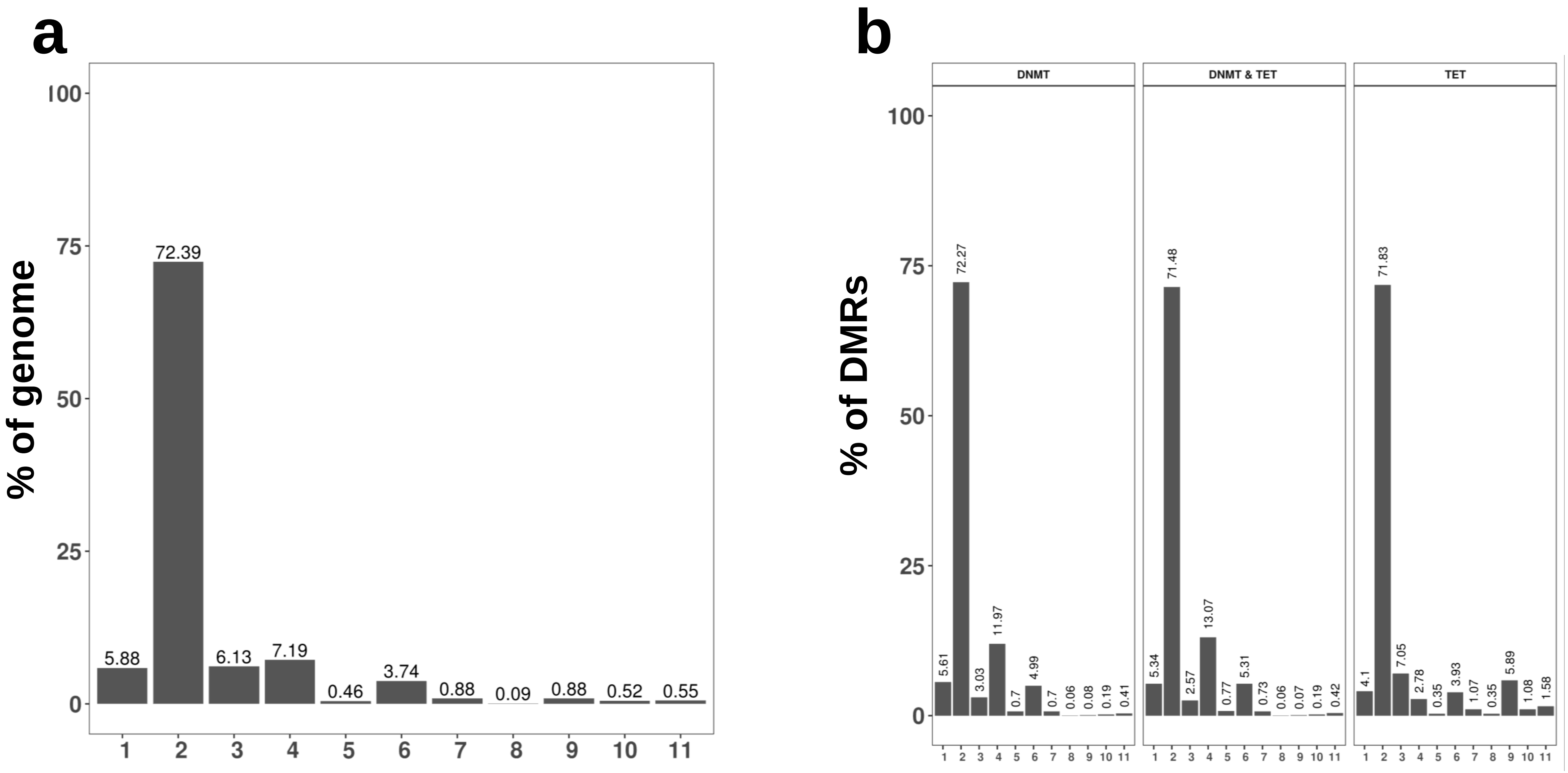

Supplementary figure 8: Distribution of chromatin states in the reference genome (a) and distribution od DMRs in the respective chromatin states (b). Chromatin states defined in Supp. fig. 7.
